# Employment in the Ecuadorian cut-flower industry and the risk of spontaneous abortion

**DOI:** 10.1186/1472-698X-9-25

**Published:** 2009-10-08

**Authors:** Alexis J Handal, Sioban D Harlow

**Affiliations:** 1 Master in Public Health Program, Department of Family and Community Medicine, University of New Mexico School of Medicine, Albuquerque, NM, USA; 2 Department of Epidemiology, University of Michigan School of Public Health, Ann Arbor, MI, USA

## Abstract

**Background:**

Research on the potentially adverse effects of occupational pesticide exposure on risk of spontaneous abortion (SAB) is limited, particularly among female agricultural workers residing in developing countries.

**Methods:**

Reproductive histories were obtained from 217 Ecuadorian mothers participating in a study focusing on occupational pesticide exposure and children's neurobehavioral development. Only women with 2+ pregnancies were included in this study (n = 153). Gravidity, parity and frequency of SAB were compared between women with and without a history of working in the cut-flower industry in the previous 6 years. Logistic regression analysis was conducted to assess the relation between SAB and employment in the flower industry adjusting for maternal age.

**Results:**

In comparison to women not working in the flower industry, women working in the flower industry were significantly younger (27 versus 32 years) and of lower gravidity (3.3 versus 4.5) and reported more pregnancy losses. A 2.6 (95% CI: 1.03-6.7) fold increase in the odds of pregnancy loss among exposed women was observed after adjusting for age. Odds of reporting an SAB increased with duration of flower employment, increasing to 3.4 (95% CI: 1.3, 8.8) among women working 4 to 6 years in the flower industry compared to women who did not work in the flower industry.

**Conclusion:**

This exploratory analysis suggests a potential adverse association between employment in the cut-flower industry and SAB. Study limitations include the absence of a temporal relation between exposure and SAB, no quantification of specific pesticides, and residual confounding such as physical stressors (i.e., standing). Considering that approximately half of the Ecuadorian flower laborers are women, our results emphasize the need for an evaluating the reproductive health effects of employment in the flower industry on reproductive health in this population.

## Background

Growing pressure from globalization on traditional agricultural production has led to a greater presence of large-scale corporate agriculture in many low income countries. According to the World Health Organization (WHO)[1], about half of the increase in pesticide use in developing countries that has occurred in the past several decades has been through use in large-scale agricultural industry. Because young women of reproductive age comprise an increasing proportion of the paid labor force within global agricultural industries more attention is warranted regarding potential adverse effects of occupational exposure to pesticides on reproductive health of these working women.

Comprehensive reviews by Sever et al. and Arbuckle et al. address the inconsistent and often controversial epidemiological evidence on the reproductive effects of occupational pesticide exposure, particularly in regard to fetal loss [2,3]. Even more limited is the available data on the reproductive toxicity of occupational pesticide exposures in agricultural populations residing in developing countries. Restrepo et al. assessed reproductive outcomes among women occupationally exposed to pesticides while employed in the Colombian cut-flower industry and found a moderately increased risk of spontaneous abortion among flower workers [4]. More recently, Crisostomo et al. reported an increased risk of spontaneous abortion among conventional pesticide users compared to integrated pest management (IPM) users in the Philippines [5]. In a study of Spanish greenhouse workers and their wives, an increased risk of spontaneous abortion was found for the wives of the high-exposure sprayers (RR = 3.14; 95% C.I. 1.25, 7.88) [6]. Some evidence exists that suggests paternal occupational exposure may influence pregnancy loss, either through genetic or biological (contaminated seminal fluid) mechanisms or via contaminated clothing or equipment [7-12].

As is true in many parts of the world, agriculture plays a critical role in the livelihood of much of the Ecuadorian population. Currently, cut-flowers are the third largest export, following petroleum and bananas. Pesticide use in this industry is widespread. According to research conducted by the Centro de Estudios y Asesoría en Salud (CEAS) in Quito, Ecuador, the most commonly used classes of pesticides in this industry are OPs, carbamates, and dithiocarbamates [13]. Some examples of the pesticides used in this industry include: mancozeb, methylbromide, captan, carbofuran, malathion, and diazinon. In the Ecuadorian cut-flower industry, over half of the workers are women of reproductive age, making assessment of reproductive risks in this industry a priority.

In 2003, we conducted an investigation on the potential associations between occupational and environmental pesticide exposure and delayed neurobehavioral development in infants and young children residing in a flower growing region of northern Ecuador [14-16]. In that study, we found that even though flower workers reported higher wages compared to other workers in the region and better access to health care and daycare, there was some aspect of their work, particularly during pregnancy, which adversely affected their child's development [16]. Using data from this study, the present analysis explores whether an association exists between reports of spontaneous abortions and work in the flower industry among the mothers participating in that study.

## Methods

### Study Population

The study population has been previously described in detail [13]. Briefly, the EcoSalud Project (CEAS/IDRC), launched in 2001 after local community leaders and members raised concerns about potential health problems among workers in the cut-flower industry and in community residents, investigates the impact of the cut-flower industry in a northeastern highland region of Ecuador. As a component of the epidemiologic aspect of the EcoSalud project, our 2003 study focused on neurobehavioral development in infants and young children in the region.

The study population was drawn from three communities selected based on potential exposure status and on having sufficient ties between CEAS and community leaders to ensure accessibility to the community. Communities A and B were at lower altitudes and likely to have higher environmental and occupational pesticide exposures given their proximity to the cut-flower industry: Community C was at a higher altitude and likely to have lower exposure as residents were less likely to work in the flower industry and lived a great distance from the flower plantations.

We conducted a census in each community to construct the sampling frame. Mothers with any children ages 3 to 61 months and who had been living in the communities for at least a year were eligible to participate. In total, 219 mothers (91% of those eligible) participated. Mothers had to have at least one child to be eligible for the neurobehavioral development study. Therefore mothers who reported only one pregnancy (n = 64) were excluded from this analysis as mothers with only one pregnancy ending in a pregnancy loss were not included in the 2003 study sample. We also excluded two women who did not provide information on reproductive history, leaving 153 eligible for this analysis.

Approval for this project was obtained from the Institutional Review Board (IRB) at the University of Michigan as well as from CEAS in Ecuador. Informed consent was obtained from the participating mothers.

### Exposure Assessment

Data on maternal occupational history was obtained through a questionnaire administered to the mother by a trained interviewer. Maternal occupation in the flower industry was assessed with two main variables: 1) occupation in the flower industry in the previous six years (yes, no); and 2) number of years spent working in the flower industry over the previous six years (total years).

### History of SAB

Reproductive histories were obtained by questionnaire for each mother. Data on the total number of live births, stillbirths, SAB, and induced abortions was obtained. Gravidity was assessed as a continuous variable. Gravidity, accounting for reported SAB, was also assessed (≥ 2 pregnancies and no history of SAB, ≥ 2 pregnancy and a history of SAB). Mothers were also asked whether she had ever tried to get pregnant, but could not for one year or more (yes, no).

### Covariates

Maternal and sociodemographic characteristics included maternal age and education level, mother's ethnicity, marital status, and monthly household income in U.S. dollars ($0-150, $151-250, or > $250). Maternal age was examined as a continuous variable and as a dichotomous variable based on the median age in the sample (≤ 25 years old, > 25 years old) and based on the existing literature (< 30 years old, ≥ 30 years old). Maternal education, categorized as none/partial primary, completed primary school, or partial/completed high school, was used to assess education level and as a proxy for literacy. Mother's educational level and her ability to read (yes/no) were correlated (r = 0.52), as were mother's educational level and her ability to write (yes/no) (r = 0.54).

### Statistical Analysis

The mother's reproductive health and socio-demographic characteristics were compared across maternal occupation status categories. Reported history of SAB was initially assessed as a continuous variable and was then assessed as a dichotomous variable (yes, no).

Logistic regression models were constructed for reported history of SAB (y/n) to assess the effect of maternal occupation in the flower industry during the previous six years, after controlling for potential confounders. One woman who reported an induced abortion was excluded in the analysis. Several methods were employed in assessing potential confounders: 1) inclusion of variables based on what has been cited in the literature; 2) inclusion of variables that were significantly associated with both maternal occupation in the flower industry in the previous six years and reported SAB; 3) inclusion of variables that changed the beta coefficient by at least 10%. Data were entered into SPSS 11.5 (SPSS Inc., Chicago, IL, USA) and were analyzed in SAS Version 8 (SAS Institute Inc., Cary, NC, USA).

## Results

Maternal characteristics are presented in Table 1 stratified by whether or not mothers had worked in the flower industry and by whether or not they had reported SAB. Thirty of the 153 mothers in our study sample (20%) reported a previous SAB. These mothers tended to be younger, however, not significantly different (mean 28.5 versus 29.2 years). A higher frequency of mothers who reported SAB self-identified as Indigenous (87% versus 74%), reported being married (77% versus 65%), and reported a slightly higher monthly household income. Educational level did not differ significantly between those who reported a history of SAB and those who did not. Mothers who reported a history of SAB reported slightly fewer live births (3.0 versus 3.7) and a higher gravidity (4.1 versus 3.7), though not significantly higher.

**Table 1:** Study Characteristics by Reported SAB and by Work in the Flower Industry in the Previous Six Years (n = 153), Ecuador, 2003

Study Characteristics	Reported History of SAB		Worked in the flower industry	
	No (n = 123)	Yes (n = 30)	No (n = 67)	Yes (n = 86)
Mother's Age (mean, SD)	29.2 (6.8)	28.5 (6.7)	31.6 (7.9)	27.1 (4.9)*
	N (%)			
Mother's Age				
≤ 25 years	42 (34.1)	14 (46.7)	20 (29.9)	36 (41.9)
> 25 years	81 (65.9)	16 (53.3)	47 (70.1)	50 (58.1)
Mother's Age				
< 30 years	76 (61.8)	18 (60.0)	32 (47.8)	62 (72.1)*
≥ 30 years	47 (38.2)	12 (40.0)	35 (52.2)	24 (27.9)
Ethnicity of Mother				
Indigenous	90 (74.4)	26 (86.7)	56 (84.9)	60 (70.6)*
Mestizo/White	31 (25.6) missing = 2	4 (13.3)	10 (15.1) missing = 2	25 (29.4)
Marital Status				
Married	80 (65.0)	23 (76.7)	47 (70.2)	56 (65.1)
Free Union	30 (24.4)	4 (13.3)	12 (17.9)	22 (25.6)
Single/Separated/Widowed	13 (10.6)	3 (10.0)	8 (11.9)	8 (9.3)
Mother's Education Level				
None or partial primary	43 (35.0)	8 (26.7)	28 (41.8)	23 (26.7)
Completed primary school	66 (53.7)	17 (56.7)	31 (46.3)	52 (60.5)
Partial or completed high school	14 (11.3)	5 (16.7)	8 (11.9)	11 (12.8)
Monthly Household Income				
$0-150	60 (49.2)	9 (30.0)	40 (60.6)	29 (33.7)*
$151-250	36 (29.5)	10 (33.3)	16 (24.2)	30 (34.9)
> $250	26 (21.3) missing = 1	11 (36.7)	10 (15.2) missing = 1	27 (31.4)
*Reproductive History*				
Total Births (mean, SD)	3.8 (2.1)	3.1 (2.3)	4.4 (2.5)	3.0 (1.6)*
Live births (mean, SD)	3.7 (2.1)	3.0 (2.3)**	4.4 (2.5)	3.0 (1.5)*
Gravidity (mean, SD)	3.7 (2.1)	4.1 (2.4)	4.5 (2.6)	3.3 (1.5)*
Gravidity:	N (%)			
2+ pregnancies & no history of SAB	--	--	59 (88.1)	64 (74.4)*
2+ pregnancies & history of SAB	--	--	8 (11.9)	22 (25.6)

* p ≤ 0.05, ** p < 0.10

A total of 86 of mothers with at least 2 pregnancies reported working in the flower industry in the previous six years (56%). These mothers were significantly younger (27.1 vs. 31.6 years), more commonly self-identified as Mestizo/White (29% versus 15%), and had a higher reported level of education and significantly higher reported monthly household income (> $150/month versus ≤ $150/month). Women working in the flower industry reported significantly fewer live births (3.0 versus 4.4) and significantly lower gravidity (3.3 versus 4.5).

Among those mothers with a gravidity of 2 or more, a higher frequency of those reporting at least one SAB reported previous employment in the flower industry (26% versus 12%). A majority of the women with 2 or more pregnancies and a reported history of SAB had worked 4 to 6 years in the flower industry (60%) (Table 2).

**Table 2:** Reported History of SAB Stratified by Gravidity for Years Worked in the Flower Industry in the Previous Six Years, Ecuador (n = 153), 2003

	Time spent working in the flower industry in the past 6 years*						
	
	None	**1 year**^a^	**2 years**^b^	**3 years**^c^	**4 years**^d^	**5 years**^e^	**6 years**^f^
Reported History of SAB by gravidity	N (%)						
≥ 2 pregnancies & no history of SAB	59 (48.0)	13 (10.6)	2 (1.6)	9 (7.3)	12 (9.8)	12 (9.8)	16 (13.0)
≥ 2 pregnancies & history of SAB	8 (26.7)	0 (0.0)	1 (3.3)	3 (10.0)	5 (16.7)	6 (20.0)	7 (23.3)

* p = 0.04

^a ^≤ 12 months; ^b ^> 12 to ≤ 24 months; ^c ^> 24 to ≤ 36 months; ^d ^> 36 to ≤ 48 months; ^e ^> 48 to ≤ 60 months; ^f ^> 60 to ≤ 72 months

The odds of reporting a history of SAB were approximately 2.6 times for those mothers who reported working in the flower industry in the previous six years as compared to those women who did not, (Table 3) controlling for maternal age (95% CI: (1.03, 6.73). The odds of reporting a history of SAB increased to approximately 3.4 for mothers who reported working in the industry for more than three of the previous 6 years, as compared to mothers who did not work in the flower industry, after controlling for maternal age (95% CI: (1.27, 8.83).

**Table 3:** Adjusted Regression Models for Reported History of SAB for Women who worked in the Flower Industry in the Previous Six Years (n = 153), Ecuador, 2003

	Worked in the Flower Industry Anytime during the Previous Six Years	
Reported History of SAB (n = 30)	OR^a^	95% CI

	2.63	(1.03, 6.73)

	Worked in the Flower Industry ≥ 4 years during the Previous Six Years	

Reported History of SAB (n = 30)	OR^a^	95% CI

	3.35	(1.27, 8.83)

^a ^adjusted for maternal age

## Discussion

This is the first study of the association between spontaneous abortion and maternal occupation in the export flower industry conducted in Ecuador. The findings from this exploratory analysis suggest a possible association between working in the Ecuadorian flower industry and risk of SAB, with women who had worked longer periods of time in the industry also having a higher risk of having had a SAB, even after controlling for maternal age. There are several potential explanations for our findings.

Research has pointed to some possible mechanisms that may explain the effects of pesticide exposure on reproductive toxicity. Organophosphate pesticides may increase the frequency of sperm sex null aneuploidy, which may increase the risk of spontaneous abortion [9]. Others have suggested a possible link between contaminated seminal fluid transfer to the mother and adverse developmental outcomes [17]. Certain chemicals such as thalidomide and cocaine bind directly to the sperm [18]. Contaminated clothing brought to the home may contribute to maternal exposure during her pregnancy, [19-21]. however, it is uncommon for the flower workers to take home their work clothes and equipment. Finally, there is some evidence suggesting a disruption of hormonal function in the female from exposure to pesticides [22].

Another possible explanation for our preliminary findings is overexertion or high physical strain associated with employment in the cut-flower industry. Previous research has suggested an association between physical strain and risk of pregnancy loss and fetal death, with a higher risk of pregnancy loss for those women with a history of spontaneous abortion [23-25]. The primary job responsibilities for the female Ecuadorian flower workers include either harvesting the flowers within a greenhouse setting or cleaning and packaging the flowers in a warehouse setting. In both instances, the workers are on their feet the majority of the day. In our larger neurodevelopmental study, 52% of the mothers who had worked in the flower industry during their pregnancy with the participating child reported working more than 45 hours per week and 67% of those mothers also reported working 6 or more months total during the pregnancy. The physical strain of this type of work along with the heat and exhaustion that can occur in a greenhouse setting may contribute to reproductive toxicity in this population. Further examination of this issue is warranted.

This exploratory secondary data analysis has several important limitations. The primary limitation is the lack of a temporal relation between the exposure and the outcome of interest, pregnancy loss. We asked the mothers about any history of pregnancy loss that had occurred in the previous six years. We were not able to determine if the pregnancy loss had occurred prior to or during her employment in the flower industry. The average age of the mothers who reported working in the flower industry was approximately 27 years old and over one third of these mothers had worked more than 3 years in flower industry. Although SAB is associated with increased maternal age, our population consisted of young women with a proportion of these women having reported working in the flower industry a substantial proportion of their reproductive age.

We did not have an actual measure of exposure other than history of maternal occupation in the flower industry so we are unable to separate out effects that might have been related to physical strain, stress, or heat from effects from pesticide exposure. Furthermore, relying on an indirect exposure measurement (i.e., history of maternal occupation in the flower industry), which does not detail the specific potential pathways of pesticide exposure, may lead to exposure misclassification. We also did not have information on the type or quantity of pesticides used domestically, another potential source of measurement error. Future investigations should incorporate the use of biomarkers and environmental sampling.

The sample size was limited for this exploratory analysis and was especially limited in terms of assessing paternal exposures. In this population, data was missing on 20% of fathers and only fathers' current work status was assessed. Thus we could not assess risk associated with potential paternal occupational exposures. It would be important to consider the father's exposure, given the data available that suggests a male-mediated role in reproductive toxicity.

Cigarette smoking and alcohol use during pregnancy may contribute to pregnancy loss [26,27]. We did not have this information for any pregnancy that ended in a loss. Also, as described above, we had information about the mother's work hours during her index pregnancy for the neurobehavioral study, but not for any pregnancy that would have ended in a loss. Data on physical strain and other important lifestyle variables should be obtained in any future investigation in this population.

Another important limitation common in studies on SAB is recall bias [28]. If the loss occurred some time ago, the time difference could influence the woman's recall. In our study, however, the mothers were fairly young so this may not be as relevant. Gestational age at the time of the loss can also influence recall. If a loss occurs at an early gestational age it may be less likely that the woman knew she was pregnant. In our population, the observed effect could be stronger if the working mothers have many early losses that they did not recognize as pregnancy losses. Finally, it is possible that the women working in the flower industry were sensitive to the difficult working conditions and may have recalled more losses because of their perceptions. However, because the overall study that the mothers were participating in was focused more on the development of the child, it may be more likely that the mother would not have been focused on her reproductive history as an outcome from pesticide exposure, thus not affecting her recall.

## Conclusion

We found an excess of reported spontaneous abortion among women working in the Ecuadorian cut-flower industry. We are unable, at this time, to distinguish what caused this excess. However, this report is consistent with reports focusing on flower workers in other parts of the world. Larger studies of this industry, with more detailed exposure assessment and better information on confounders are needed.

A large number of women of reproductive age work in the Ecuadorian flower industry where exposure to pesticides is highly probable, the hours are long and pregnant women are likely to spend much of the work day standing. Although findings from this exploratory analysis should be interpreted with caution, given the lack of research in this area, the findings from this preliminary analysis emphasize and highlight the need for an extensive investigation into the effects of employment in the flower industry on reproductive health in this population.

## Competing interests

The authors declare that they have no competing interests.

## Authors' contributions

Both authors were involved in the design and analysis of the 2003 study and the development of the present study. AJH preformed the statistical analysis and drafted and wrote the manuscript, with contributions from SDH. Both authors read and approved the final manuscript.

## Pre-publication history

The pre-publication history for this paper can be accessed here:

http://www.biomedcentral.com/1472-698X/9/25/prepub
